# Multi-Stage Cold Forging Process for Manufacturing a High-Strength One-Body Input Shaft

**DOI:** 10.3390/ma14030532

**Published:** 2021-01-22

**Authors:** A Ra Jo, Myeong Sik Jeong, Sang Kon Lee, Young Hoon Moon, Sun Kwang Hwang

**Affiliations:** 1 Mechanical Components and Materials R&D Group, Korea Institute of Industrial Technology, Daegu 42994, Korea; ooo7208@kitech.re.kr (A.R.J.); msjeong@kitech.re.kr (M.S.J.); sklee@kitech.re.kr (S.K.L.); 2 Department of Mechanical Engineering, Pusan National University, Busan 46241, Korea; yhmoon@pusan.ac.kr

**Keywords:** input shaft, multi-stage cold forging process, shrink fit, torsion test, fatigue test

## Abstract

A multi-stage cold forging process was developed and complemented with finite element analysis (FEA) to manufacture a high-strength one-body input shaft with a long length body and no separate parts. FEA showed that the one-body input shaft was manufactured without any defects or fractures. Experiments, such as tensile, hardness, torsion, and fatigue tests, and microstructural characterization, were performed to compare the properties of the input shaft produced by the proposed method with those produced using the machining process. The ultimate tensile strength showed a 50% increase and the torque showed a 100 Nm increase, confirming that the input shaft manufactured using the proposed process is superior to that processed using the machining process. Thus, this study provides a proof-of-concept for the design and development of a multi-stage cold forging process to manufacture a one-body input shaft with improved mechanical properties and material recovery rate.

## 1. Introduction

Various automobile parts are manufactured by several processes. Among them, products that are manufactured by machining have highly precise dimensional shape and surface condition. However, this method has several problems, such as low material efficiency and the chip removal method is inefficient. Hence, the automotive industry is now focused on replacing the machining process with a metal forming process to obtain higher mechanical properties, productivity, and material recovery [1].

The input shaft is an important automotive part of the motor-driven power steering (MDPS) system. Its main function is to transmit the driving force and torque to the universal joint. Hence, it requires superior mechanical properties and geometric tolerance. Generally, the input shaft is manufactured by machining because its length exceeds 200 mm and it has a complicated shape. Another fabrication process of the input shaft includes separately manufacturing its upper and lower parts by cold forging and then combining them. However, these processes could not satisfy the required geometric tolerance and avoid defects, such as dropouts and assembly.

Forging has steadily gained traction as the primary metal forming process as its processing parameters, such as working temperature and environment, can be adjusted to produce the required material properties. Particularly, cold forging that is performed below the recrystallization temperature has excellent dimensional accuracy and produces excellent mechanical properties. This is due to the dislocations generated by the high flow stress. However, this high flow stress requires a high load and only a limited variety of shapes can be produced. Hence, the cold forging process alone, or one combined with hot forging, has been previously used [2,3,4,5,6]. Fujikawa et al. [7] described a method for combining warm and cold forging to manufacture constant-velocity joints and spline shafts for automobiles. Using this combination, complex and high-load products can be easily manufactured. However, the addition of heat treatment and pickling treatment in the process makes it complicated and expensive. Consequently, it is important to investigate other methods that preserve the simplicity and cost-effectiveness of the process.

Multi-stage cold forging without heat treatment has been employed by researchers. This process distributes the load and increases the accuracy of the shape. Pang et al. [8] used multi-stage cold forging for manufacturing hollow power-transmission long shafts. Ku et al. [9] successfully replaced traditional multi-stage warm forging with multi-stage cold forging to produce constant-velocity joints. The products manufactured revealed excellent mechanical properties. Thus, multi-stage cold forging may be a prime candidate for efficient manufacturing of input shafts. In addition, it is important to predict the results of the process and die design to increase the die life in the process design. This is usually performed using finite element analysis (FEA). McCormack and Monaghan [10] proposed the most suitable die design for manufacturing hexagon head bolts by analyzing the forming results with respect to the head angle using FEA. Soranansri et al. [11] designed a die with an appropriate shrink fit, which was used to improve die life, to manufacture bevel gears using forging.

In process design, the prediction of internal fracture in the product is also an important factor. For volumetric forming process, internal fracture can be predicted by the ductile fracture equation. Cockcroft and Latham [12] proposed a ductile fracture equation for plastic strain as a function of the maximum principal stress. Chen et al. [13] and Oh et al. [14] predicted ductile fractures in the extrusion and drawing processes by combining the Cockcroft–Latham ductile fracture equation with FEA. Ductile fracture occurs when the hydrostatic stress (mean stress) is non-negative or the critical damage value is exceeded at the center of the material [15,16]. The Cockcroft–Latham equation [12] and the hydrostatic equations can predict internal fractures, such as chevron cracks appearing in long products. Lee et al. [17,18] synthetically produced a high-strength bolt that is elongated in the extrusion direction, and used a multi-stage cold forging process to improve its mechanical properties with a shrink fit and a ductile fracture equation. Thus, multi-stage cold forging is the most appropriate process to manufacture high-strength products with high productivity and material recovery. Hence, it is important to design a die with a suitable shrink fit to increase die life using the ductile failure equation to predict the internal defects of a product. 

This study provides a proof-of-concept for a multi-stage cold forging process to manufacture high-strength one-body input shafts of long lengths without separate parts. The process comprises six stages and was designed using FEA simulations. The value of the shrink fit was adjusted to improve the die life and predict the die fracture. The Cockcroft–Latham equation and hydrostatic stress analysis were used to predict the ductile fracture. Based on the analysis, the dies were produced and forging was carried out. Various experiments were conducted to compare this shaft with that produced using a conventional machining process. Tensile and hardness tests were performed to confirm the improvement of the mechanical properties. Furthermore, torsion and fatigue tests were performed to compare durability. Electron backscatter diffraction (EBSD) was also used to characterize the microstructural transformation in the material. In addition, FEA was used to compare these results with those of the process that separately manufactures the upper and lower parts. The analysis results were compared based on previous experimental results.

## 2. Materials and Methods 

### 2.1. Forming

**Figure 1:**
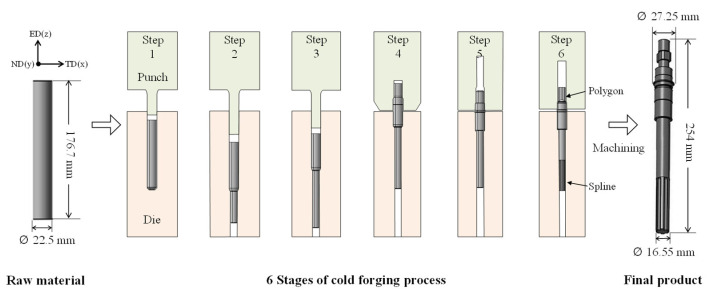
Schematic of the multi-stage cold forging process.

**Figure 2:**
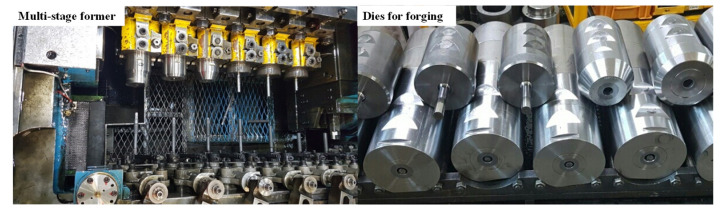
Experimental set-up for multi-stage cold forging and the dies used.

SWRCH45F steel, which is mainly used in various automobile parts, made by POSCO, was used in the study. Its analyzed chemical composition is listed in Table 1. Multi-stage forging was designed to work in six stages to produce a one-body product. The size of the raw material was determined based on the required dimensions of the final product. The diameter of the raw material was set to 22.5 mm along the diameter of the body of the final product. The length of the raw material was determined to be 176.7 mm. In this case, barreling can occur due to the long length of the product. To prevent barreling, the lower part of the length was first extruded in three steps and then the upper part was extruded in two steps. The area reduction ratio was calculated to be 47% and 45% in the upper and lower part, respectively. These values are less than the reduction ratio of the general extrusion process, which is 75% [19]. The final process design is shown in Figure 1. Step 1 is a pre-process step for the bottom extrusion. Steps 2 and 3 are for the bottom extrusion, while steps 4 and 5 are for the top extrusion. The final step, step 6, consists of forming polygons at the upper part and spline shapes at the bottom. Figure 2 shows the experimental set-up of the six-stage former (HBF-416) owned by SUNG JIN FO-MA INC and the dies used in the study. The velocity of the punch was 140 mm/s. 

### 2.2. Mechanical Characterization

**Figure 3:**
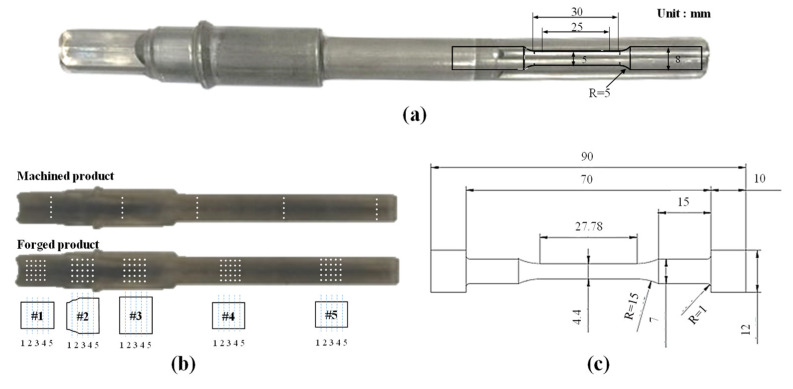
(**a**) Dimension of tensile test specimen, (**b**) data acquisition points for the Vickers micro-hardness test, and (**c**) dimensions of the specimen for the fatigue test.

Tensile tests were conducted to investigate the mechanical property changes, such as ultimate tensile strength (UTS) and elongation. The specimen was obtained from the spline part of the input shaft at the bottom and the dimensions of the specimen according to the ASTM standard (E8/E8M) are shown in Figure 3a [20]. The tensile tests were carried out in triplicate, using Zwick-Z250N, with a pre-load of 5 MPa, a constant cross-head speed of 10 MPa/s in E-modulus, and a yield point at room temperature. The properties of the shafts produced using the conventional process were used for comparison. The hardness of the input shaft was measured using a Vickers micro-hardness tester (Matsuzawa, Akita Pref, Japan) (AMT-X7FS Type B, load: 29.4 N (300 gf)) at the location shown in Figure 3b.

Torsion and fatigue tests were performed to examine the improvement in the torsional and fatigue strengths, respectively. The torsion test was conducted in two different conditions using a torsion testing machine (MET-100H) (Cowon, Seoul, Korea) at a constant speed of 100°/min at room temperature. In one condition, the specimen was tested until a fracture occurred, while in the other condition, the specimen was tested up to 200 Nm. The set torsion of 200 Nm considers a safety factor of 100 Nm, which is the maximum torque received by the MDPS [21]. To compare the deformation, a 3D laser scanner (DS-3040, error range: 6 μm) was used. Axial load fatigue tests were performed using a fatigue testing machine (ACEONE, Inchon, Korea) at room temperature to measure the fatigue limit of the machined and forged products. Figure 3c shows the dimensions of the specimens used in the fatigue test that was machined from the processed shaft according to the ASTM Standard (D7791-17) [22]. The conditions of the test are listed in Table 2. The fatigue limit was measured based on 1,000,000 cycles, which is the fatigue limit of general steel [23,24]. In addition, the fatigue test analysis was performed using ANSYS (Ansys, Canonsburg, PA, USA) using the results of the tensile and fatigue tests.

### 2.3. Microstructural Characterization

The product was cut in the normal direction (ND) plane to check for internal defects and damage, and to compare analyses and experimental results. To observe the metal flow characteristics, the specimen was hot-mounted and mechanically polished up to 1 μm using SiC papers and diamond suspensions. Macro-etching (nital 10%) was performed. The observation locations were set to four points in the final product using a stereo microscope (Carl Zeiss, Obercohen, Germany) (SteREO Discovery. V20) at 4.7× magnification. 

The microstructure evolution was observed by an EBSD, an Oxford symmetry with JEOL (JSM-7900F) (JEOL Ltd, Tokyo, Japan) [25,26]. The specimens were hot-mounted, mechanically polished by SiC paper, and mirror-finished using diamond suspensions (up to 0.25 μm) and colloidal silica. The measurement conditions of the EBSD were: an accelerated voltage of 15 kV, a probe current of 15 nA, a specimen tilt of 70°, a scan step size of 0.08 μm, and a measurement area of 79.4 × 56.9 μm^2^. A map of the kernel average misorientation (KAM) and grain boundary (GB) was obtained from the post-process program (Aztec, CHANNEL 5) (2.0, Oxford Instruments, Abingdon-on-Thames, England).

## 3. Finite Element Analysis (FEA) 

**Figure 4:**
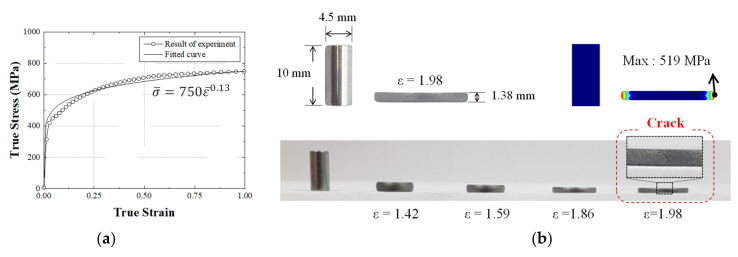
(**a**) Fitted flow stress–strain curve obtained from the compression test, (**b**) upsetting test results for various height reductions.

**Figure 5:**
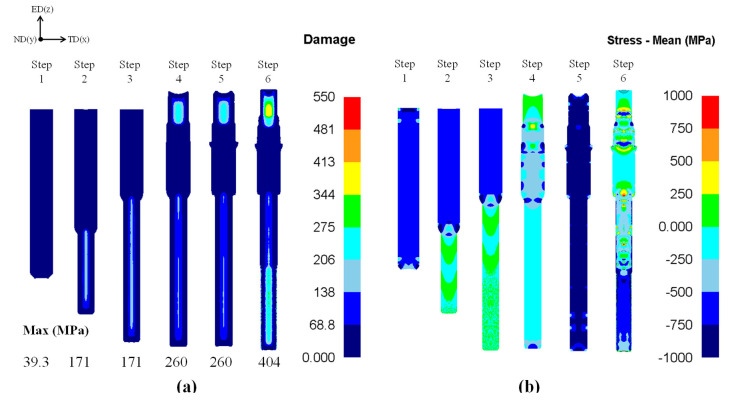
Numerical results of (**a**) ductile fracture prediction and (**b**) hydrostatic stress (mean stress).

A simulation using an FEA program is required to ensure a suitable design of the new process. Commercial FEA programs DEFORM-2D (10.0, Scientific Forming Technologies Corporation, OH, USA) and -3D (rigid plastic FEA software) (10.0, Scientific Forming Technologies Corporation, OH, USA) were used to predict the defects and damages in the products and dies. DEFORM-2D was applied to simulate steps 1 to 5 because the part produced in these steps has an axisymmetric structure. DEFORM-3D was applied to step 6 because the non-axisymmetric structure and complex shape of the part produced involved a polygon and spline. It was constructed as a one-quarter model considering its symmetrical shape. The analysis conditions are listed in Table 3. The friction coefficient between the material and the die was applied to the coulomb friction [15]. A compression test was performed to obtain the mechanical properties for the FEA and the critical damage value for the ductile fracture prediction [27]. The test was performed using a universal testing machine (Zwick-Z205N) (Zwick Roell Group, Ulm, Germany) with a constant velocity of 0.05 mm/s at room temperature. Grease was applied to the surface of the specimen to reduce friction. A stress–strain curve obtained from the compression test was applied for the ductile fracture simulation. The critical damage value of 519 MPa was obtained from the ductile fracture equation, i.e., the Cockcroft–Latham equation, as shown in Figure 4. The ductile fracture prediction and the hydrostatic stress distribution are shown in Figure 5. The ductile fracture analysis showed that the critical damage value was evenly distributed under 519 MPa for all processes. In addition, a negative hydrostatic stress was distributed in the product, which is expected to have no defects during all the processes. 

The prediction of die fracture is important in the design of the die and processes. Generally, a die with a suitable shrink fit can increase the die life and prevent die fracture. It is important to check the maximum principal stress since fracture is mainly caused by repeated tensile stresses [28,29]. The die primarily used in the industries consists of an insert, stress ring, and case for efficient use. The insert, which is located inside the die, has the highest load distribution. The stress ring is installed in the center of the die to prevent the fracture of the insert due to compressive stresses. The case is located outside the die to supplement the overall load. In this study, the materials used for the insert, stress ring, and case were tungsten carbide (WC), AISI H13, and AISI 4140, respectively. The detailed mechanical properties of each die component are listed in Table 4.

**Figure 6:**
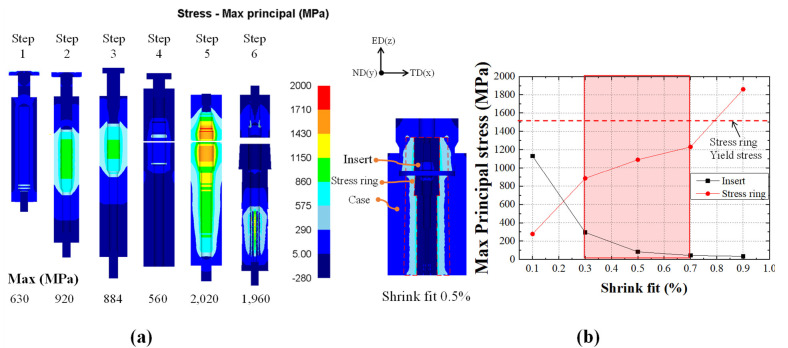
Numerical results of (**a**) maximum principal stress distributions obtained from die stress analysis and (**b**) comparison of the die stress among various shrink fits.

It is important to find the appropriate shrink fit because the die can be reinforced depending on the shrink fit of the stress ring. An insert die analysis was performed, as shown in Figure 6a. The yield strength of the insert die was less than 2683 MPa, but it had a relatively high value in steps 5 and 6. Step 6, which has a high absolute stress, was used to find the appropriate shrink fit. As shown in Figure 6b, the principal stress of the insert and stress ring is evenly distributed when the shrink fit is in the range of 0.3–0.7%. It is shown that an appropriate shrink fit can prevent die fracture and improve die life. In this study, a 0.5% shrink fit was applied.

## 4. Results and Discussion

**Figure 7:**
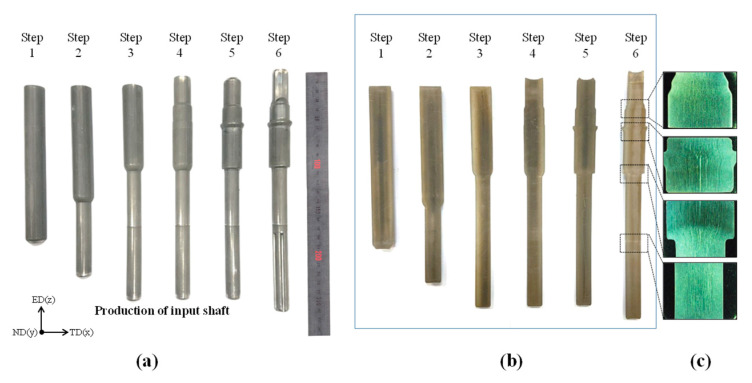
View of the (**a**) surface of the product, (**b**) interior of the product, and (**c**) metal flow of the produced product.

Based on the FEA, a die with a 0.5% shrink fit was manufactured and installed on the former, as previously shown in Figure 2. The product was cross-sectioned along the ND plane to observe the surface and interior. We confirmed that there was no damage on the surface and within the product, as shown in Figure 7. Metal flow was observed without any defects owing to its symmetry and density, and without kinks and breaks, as shown in Figure 7c. Table 5 shows the material recovery rate of the processes. The recovery rates of the machined and forged products are 31.3% and 80.3%, respectively. The material recovery rate of the forged product is approximately 2.56 times higher than that of the conventional machining process. The experimental and numerical results confirmed that the proposed process successfully manufactured a one-body input shaft with an improved material recovery rate and no defects. 

**Figure 8:**
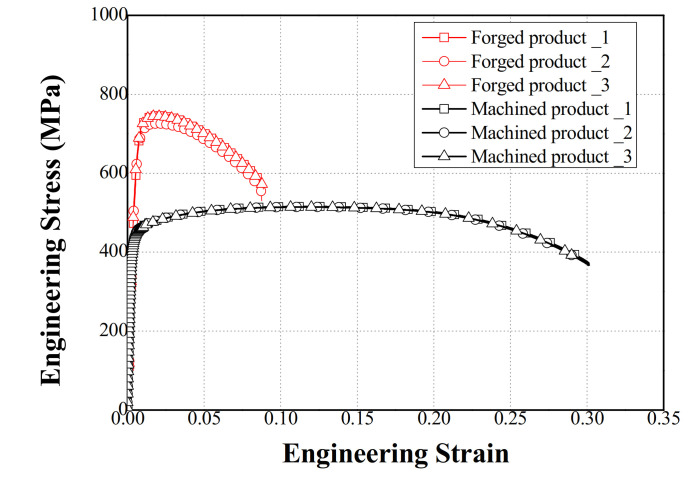
Comparison of the tensile test results for the forged and machined products.

**Figure 9:**
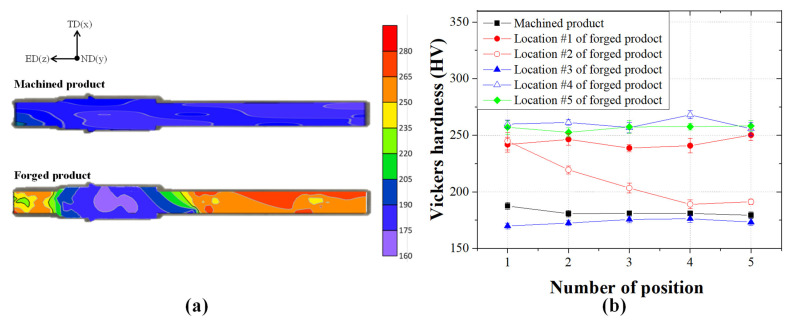
(**a**) Contour plots and (**b**) comparison graph of the Vickers hardness test results for the machined and forged products.

As shown in the stress–strain curves in Figure 8, the UTS of the machined product was 500 MPa; conversely, that of the forging product was 750 MPa. Thus, the multi-stage cold forging process can produce an input shaft with higher strength than that with the machining process, as confirmed by the approximately 50% improvement in the UTS. As shown in Figure 9, the hardness of the forged product improved by approximately 45% compared with the machined products. The polygonal (#1) and spline (#5) regions with high forming effects show the relatively high hardness values. From these results, the proposed multi-stage cold forging process can manufacture one-body input shafts with improved mechanical properties.

**Figure 10:**
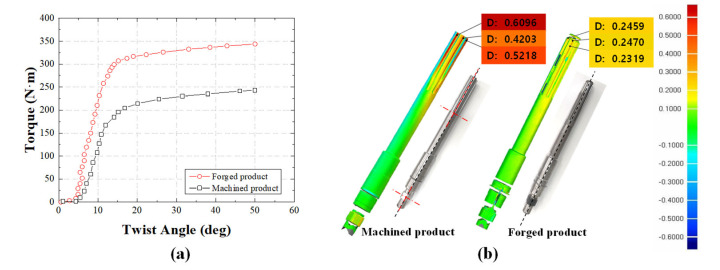
(**a**) Torque–angle curves for comparison of the machined and forged products from the torsion test and (**b**) results of the 3D scan obtained before and after the torsion test.

The torsion test was conducted until a fracture occurred in the product, as shown in Figure 10a. The torque of the machined product was 125, 215, and 230 Nm at the twisted angles of 10°, 20°, and 30°, respectively, while that of the forged product was 225, 320, and 330 Nm, respectively. The torque of the forged product was approximately 100 Nm higher than that of the machined product at the same twisted angle. A torque of 200 Nm was applied to check the dimensional change of the input shaft and the results were numerically confirmed by a 3D laser scanner (Laser Design Inc., Minneapolis, MN, USA). As shown in Figure 10b, the maximum deformation of the machined product under twisting was 0.6096 mm, while that of the forged product was 0.2470 mm. Under identical load, the deformation of the machined product was approximately 2.47 times higher than that of the forged product. Similar to the results of the tensile and hardness tests, the input shaft manufactured by the forging process has higher torsion properties and deformation resistance against torsion than the shaft produced using the machining process.

**Figure 11:**
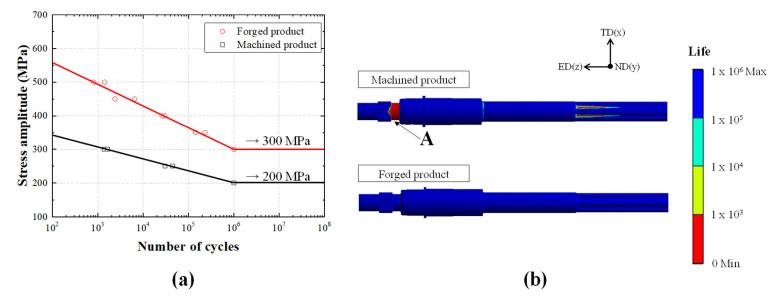
(**a**) Comparison of the machined and forged products based on their Stress amplitude–Number of cycles curve obtained via the fatigue test and (**b**) results of the fatigue analysis.

The fatigue test results are shown in Figure 11a. The fatigue limit of the machined product was 200 MPa, while that of the forged product was 300 MPa, which is approximately 50% higher. The fatigue test results were applied for additional FEA to analyze fatigue life with the 100 Nm torque, which is mainly received from the MDPS and is distributed in the extrusion direction of the polygonal area. As shown in Figure 11b, we confirmed that a fracture occurs at a measurement less than 1000 cycles in the A-position of the machined product, whereas a fracture did not occur on the forged product under 1,000,000 cycles. To increase the fatigue life of the machined input shaft, an additional high-frequency heat treatment should be applied on the A-position. These results showed that the fatigue limit of the forged input shaft increased by 100 MPa compared to the machined input shaft, thereby significantly improving the fatigue life without an additional heat treatment process. 

**Figure 12:**
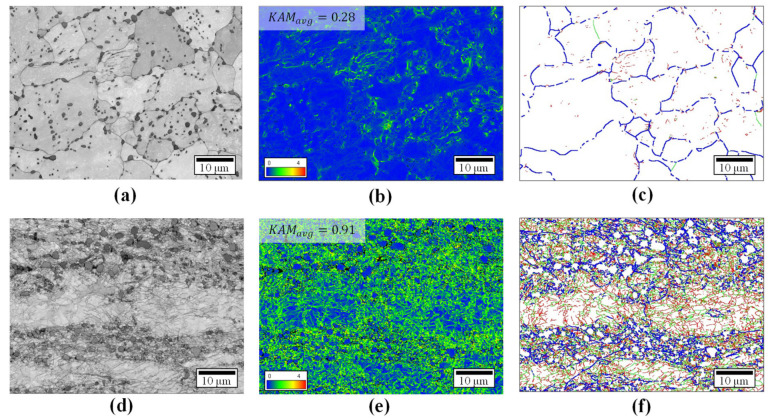
Comparison of band contrast map (**a**,**d**), KAM map (**b**,**e**), and GB map (**c**,**f**) of LAGBs (2° ≤ *θ* < 5°, red lines and 5° ≤ *θ* < 15°, green lines) and HAGBs (15° ≤ *θ*, blue lines): (**a**–**c**) raw material and (**d**–**f**) forged product.

Figure 12 shows the band contrast map, KAM map, and GB map of the raw material and forged products. The raw material was produced by hot rolling and the specimen exhibits typical medium carbon steel characteristics, such as ferrite and pearlite microstructures (Figure 12a). For the specimen that deformed by the multi-stage cold forging process, the grains were elongated in the extrusion direction (Figure 12d). KAM is the average of the local orientation differences between individual measuring points and adjacent measuring points. The KAM value increases as the dislocation density or internal-strain energy accumulated in the specimen increases. The average KAM values of the raw material and forged products were 0.28 (Figure 12b) and 0.91 (Figure 12e), respectively. For the forged products, the average value increases by 225%, indicating the accumulation of internal strain energy and dislocation density. If the misorientation angle of GB is higher than 15°, it is termed as a high-angle grain boundary (HAGBs, blue line), and if it is less than 15°, it is termed as a low-angle grain boundary (LAGBs, red (2–5°) and green line (5–15°). Almost all the grains of the raw material were equiaxed ferrite grains surrounded by a HAGB (Figure 12c). The grain of the forged product was divided by the formation of LAGBs and HAGBs due to the large plastic deformation (Figure 12f). 

**Figure 13:**
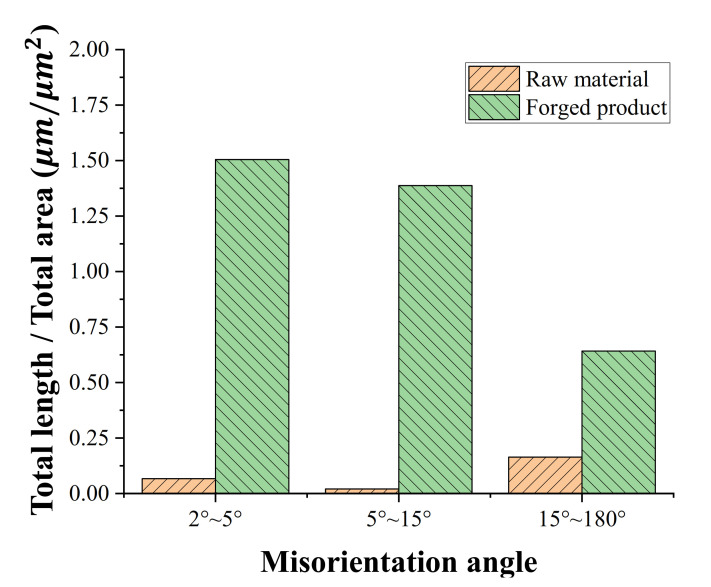
Comparison of areal fraction of misorientation angles for raw material and forged product.

Figure 13 shows the areal fraction (length of the misorientation angle divided by the measured area, μm/μm^2^), the change in the LAGBs and HAGBs during the deformation for a quantitative comparison [30,31]. The LAGBs (2–15°) and HAGBs (15–180°) fraction values of the raw material were 0.09 and 0.16 μm^−1^, respectively, while those of the forged product were 2.89 and 0.64 μm^−1^, respectively. This confirmed that the fraction value of the forged product was higher than that of the raw material. According to several researchers [32,33], the grain refinement sequence is followed by a dislocation accumulation, a formation of LAGBs, an increase in misorientation angles, an absorption of dislocations at HAGBs, and an establishment of a steady state. Therefore, from these EBSD results, a multi-stage cold forging process could be effective in the grain refinement of input shafts and in efficiently producing high-strength products with more LAGBs and HAGBs.

**Figure 14:**
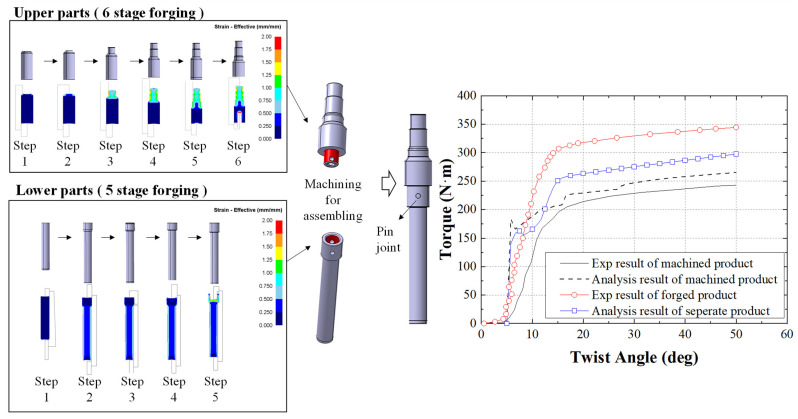
Numerical analysis of the separate input shaft process and the comparison of torque–angle curves obtained from numerical and experimental torsion test.

An additional numerical study was carried out as shown in Figure 14 to compare the separately manufactured input shaft consisting of upper and lower parts, and the one-body input shaft. The multi-stage cold forging process was designed and the same material flow-stress (Figure 4a) was applied. From the results, the separate input shaft process could manufacture the product without defect. For separate manufacturing, the upper and lower parts were machined for assembly and then joined with a pin (AISI D2) after the forging process. We used the numerical torsion test to investigate and compare the torque–angle curves of the processes. According to the results obtained from the machined product, a difference between the numerical and experimental results for the beginning of the test was observed, as shown in Figure 14. The general trend of the simulation was fairly consistent with the experimental result. Thus, the simulation condition is assumed to be reasonable for the torsion test. We observed that the torque value of the separately manufactured input shaft was higher than that of the machined input shaft and was lower than that of the one-body input shaft. From the result, the one-body input shaft manufactured by the multi-stage cold forging process is confirmed to be superior to the existing processes in terms of mechanical properties, material recovery rate, and productivity. For improving the expendability and scalability, further study could be conducted using lightweight materials to improve the implementation of the process [34].

## 5. Conclusions

In this paper, a multi-stage cold forging process was proposed to manufacture a high-strength one-body input shaft with a long length and a high material recovery rate. From the FEA and experimental results, the process was successfully designed, and the one-body input shaft could be well-manufactured with high strength, hardness, torque, and fatigue limit. The following conclusions were obtained:

1. From the FEA, it was predicted that the one-body input shaft has no external and internal defects with the Cockcroft–Latham equation and the hydrostatic stress analysis, and that the die life could be improved with a suitable shrink fit for the proposed process. The proposed process has a material recovery rate of 80.3%, which is 2.56 times higher than that of the conventional machining process.
2. The numerically designed and verified multi-stage cold forging dies were produced to verify its application in the manufacturing of a high-strength one-body input shaft. The one-body input shaft was successfully manufactured with no defects.
3. From the tensile and hardness test results, the mechanical properties improved by approximately 45–50%, and the durability obtained from the torsion and fatigue results also increased. The proposed process could be effective in the grain refinement of the input shafts and in efficiently producing high-strength products with more LAGBs and HAGBs.
4. Additional FEA was successfully carried out to compare the separately manufactured input shaft consisting of upper and lower parts and the one-body input shaft produced by the proposed process. The results confirmed that the one-body input shaft is superior to the input shafts produced by existing processes in terms of the torque value.
5. A one-body input shaft was manufactured using conventional SWRCH45F metal. Lightweight materials, such as aluminum or titanium, are increasingly applied in several parts of an electric vehicle to reduce the vehicle’s weight and improve the energy efficiency. Hence, lightweight materials can be applied during the cold forging process for manufacturing the automobile parts, such as a high-strength input shaft, to improve mechanical properties and reduce vehicle weight.

## Figures and Tables

**Table 1:** Chemical compositions of the raw material.

Material	Chemical Composition (%)				
SWRCH 45F	C	Si	Mn	P	S
	0.42–0.48	0.1–0.35	0.6–0.9	0.030 Max	0.035 Max

**Table 2:** Experimental conditions for the fatigue test.

Process Conditions	Value
Fatigue limit	1,000,000 cycles
Frequency	10 Hz
Stress ratio (σ_min_/σ_max_)	−1
Stress Amplitude of Machined product	200–300 MPa
Stress Amplitude of Forged product	300–500 MPa

**Table 3:** Process conditions for the FEA.

Process Conditions	Value
Symmetric model of DEFORM-2D	axis symmetry
Symmetric model of DEFORM-3D	quarter (90°)
Initial specimen Diameter	22.5 mm
Initial specimen Length	176.7 mm
Friction	μ = 0.055
Punch velocity	140 mm/s
Temperature of Specimen	20 °C
Temperature of Die	20 °C
Temperature of Environment	20 °C

**Table 4:** Material properties of the forging dies.

Die Material	Elastic Modulus	Yield Stress
Insert (WC)	468 GPa	2683 MPa
Stress ring (AISI H13)	215 GPa	1380 MPa
Case (AISI 4140)	205 GPa	1110 MPa

**Table 5:** Comparison of material recovery rate between the processes.

	Machined Input Shaft	Forged Input Shaft
Raw material volume (mm^3^)	180,248	70,257
Final product volume (mm^3^)	56,430	
Material recovery rate (%)	31.3	80.3

## Data Availability

New data were created or analyzed in this study. Data sharing is not applicable to this article.
